# *In Vitro* Detection of Estrogen Activity in Plastic Products Using a Sensitive Bioassay: Failure to Acknowledge Limitations

**DOI:** 10.1289/ehp.1103894

**Published:** 2011-09-01

**Authors:** William R. Kelce, Christopher J. Borgert

**Affiliations:** Exponent, Cary, North Carolina, E-mail: wkelce@exponent.com; Applied Pharmacology & Toxicology Inc. Gainesville, Florida

Yang et al. (2011) used the *in vitro* E-SCREEN assay to infer that health risks from “estrogenic” plastics can be eliminated by using their proprietary materials, processes, and products to manufacture plastics. An *in vitro* cell proliferation assay such as the E-SCREEN is a sensitive indicator of *in vitro* estrogen agonist activity and potential estrogenic activity *in vivo* (e.g., in the rat uterotrophic assay). However, *in vitro* properties may not manifest in *in vivo* activity, and neither demonstrates a health risk. Without definitive evidence that *in vivo* activity leads to adverse health effects, the results of Yang et al. are unconvincing and fail to support changing current manufacturing processes for plastics.

The value of *in vitro* and *in vivo* estrogenic assays for predicting adverse health effects is largely untested but would need to account for actual exposure levels, metabolism, distribution, excretion, and the affinity of parent compounds and metabolites for estrogen receptor binding and transcriptional activation relative to and in competition with physiological levels of potent endogenous hormones. The combined effects of these exposures would also need to be assessed in the context of dietary (e.g., milk, cheeses, vegetables, meats, and other foodstuffs) and environmental estrogens. An excellent *in vitro*/*in vivo* study of combined effects (Charles et al. 2007) showed that while relatively high levels of a putative synthetic estrogen mixture increased the estrogenic action of common dietary phytoestrogens, low levels were without effect. Thus, sensitive *in vitro* detection may not portend estrogenic effects amid the endogenous and dietary hormonal milieu.

Yang et al. (2011) made inferences about the safety of plastic food packages, but it is unfortunate that they did not use an extraction method that was approved by the U.S. Food and Drug Administration (FDA 2007). This would have improved the reliability and applicability of their results. Although food typically contacts only the inside surface of containers, Yang et al. extracted materials from 4-mm squares of cut plastic, exposing the inside, outside, and cut surfaces to the extraction medium. Substances may leach into food from the exposed surface of a plastic container but do not typically migrate through the plastic layer (Franz and Welle 2009); thus Yang et al.’s extraction method differs from FDA-approved methods and the way foods normally contact containers. Experimental error was not reported, making comparison of these results with standard methods impossible.

In the study by Yang et al. (2011), irradiation methods for simulating “stress” were not well characterized, but they appear to have involved all surfaces of the plastic squares. However, even clear plastics can filter ultraviolet (UV) rays, reducing the potential irradiation of inside container surfaces. Similarly, colorants were added to the extraction mixture; however, during the production of plastics, colorants are embedded and tightly linked. The extent to which these procedures may have confounded the data cannot be known, but the resulting tested extracts may be substantially different from residues that could enter food from plastic containers.

Yang et al. (2011) indicated that without increasing production costs, they can identify and/or have developed monomers, additives, and processing agents that lack estrogenic activity. This conclusion appears to derive from data for resins P1, P2, P3, P4, P19, and maybe P18 in their Table 3. In the text the authors noted six MCF-7 assays, but it is unclear whether a single assay was conducted for each of the six stressor and extraction combinations (microwave, UV, autoclave, saline, and ethanol) or whether the whole series was completed six times. Regardless, the authors provided no estimate of assay variance, making it difficult to differentiate real differences from experimental error. In addition, the relative safety of these new agents, particularly antiandrogenic potential, has yet to be resolved.

In conclusion, Yang et al. (2011) provided interesting observations but failed to acknowledge the significant limitations of their observations to human health risk assessment. They relied on a very limited *in vitro* screen to model a very complex system, and those reviewing the study should be aware of the limitations of the approach and the interpretation of such data.
